# Comparing Pediatric Dermatology Research Funded by the US National Institutes of Health (NIH) With the US Skin Disease Burden in Patients Under 20 Years Old

**DOI:** 10.1111/pde.70007

**Published:** 2025-08-17

**Authors:** Elyse Mackenzie, Nishita Amancharla, Stephanie Casagrande, Waasae Ayyaz, Madeline Tchack, Noah Musolff, Babar Rao

**Affiliations:** ^1^ Rutgers Robert Wood Johnson Medical School Piscataway New Jersey USA; ^2^ Rao Dermatology New York New York USA; ^3^ Center for Dermatology Rutgers Robert Wood Johnson Medical School Somerset New Jersey USA; ^4^ Department of Dermatology Weill Cornell Medicine New York New York USA

## Abstract

**Background:**

Disease burden, measured by disability‐adjusted life years (DALYs), is a helpful metric to guide research funding priorities. Pediatric dermatologic conditions significantly contribute to DALYs, yet it is unclear whether NIH funding reflects this burden.

**Objectives:**

To compare NIH‐funded dermatology research with the most burdensome skin diseases in the United States for patients under 20 years old, as measured by DALYs, and to identify mismatches between funding and disease burden.

**Methods:**

A cross‐sectional analysis was independently performed by two researchers who matched projects from the 2024 to 2025 NIH Research Portfolio Online Reporting Tools with 10 pediatric dermatology skin conditions and their respective DALYs from the Global Burden of Disease (GBD) 2021 study. NIH‐funded research projects were categorized by condition and pediatric focus, and funding allocation was compared to DALYs to evaluate alignment.

**Results:**

The NIH supported 307 grants totaling $388 million across the 10 skin conditions. Atopic dermatitis received the most funding ($62.99 million), with 47.9% of grants pediatric‐focused. Viral skin diseases, despite being second in burden, received only $2.35 million and 0 pediatric grants. Most conditions had fewer than 15% pediatric‐focused grants.

**Conclusions:**

There is a persistent mismatch between NIH funding and the burden of pediatric skin disease, both in overall funding and in the proportion of research focused on pediatric populations. Increased investment in high‐burden, underfunded conditions, particularly those lacking pediatric‐specific research, is essential.

## 1 Introduction

The 2021 Global Burden of Disease (GBD) study is part of an ongoing research initiative that measures the impact of diseases, injuries, and risk factors on global health [1]. The most recent findings, published in May 2024, examined morbidity and mortality trends from 1990 to 2021 for 288 causes of death, 371 diseases and injuries, and 88 risk factors [2]. Disease burden is measured in disability‐adjusted life years (DALYs), a composite metric combining years of life lost due to premature death and years lived with disability, where 1 DALY equals 1 lost year of healthy life [3]. The GBD allows for epidemiologic comparisons across regions and subpopulations, including pediatric patients in the U.S., enabling targeted analysis and action.

As the largest global funder of biomedical research, the National Institutes of Health (NIH) uses disease burden as a key metric in allocating funds [4]. However, funding often misaligns with burden. A landmark 1999 study found DALYs were the strongest predictor of NIH funding but explained only 39% of the variance [5]. In response, Congress directed the Institute of Medicine (IOM) to review the NIH's priority‐setting process, leading to recommendations to better incorporate disease burden [6]. A follow‐up by Gillum et al. [7] found that while NIH funding in 2006 remained associated with DALYs (*p* = 0.001), they explained just 33% of funding variation, indicating modest improvement. These findings highlight persistent disparities in NIH allocation despite prioritization efforts.

This alignment is particularly critical in pediatric dermatology, where conditions like atopic dermatitis, acne, and psoriasis significantly affect quality of life [8]. Despite their prevalence and burden, pediatric skin diseases receive disproportionately low NIH funding [9]. A 2021 cross‐sectional analysis of NIH funding versus disease burden across pediatric conditions found that dermatitis was the second most underfunded condition relative to its DALYs, receiving $75 million less than expected [9]. A 2015 study of NIH funding across all age groups revealed similar trends: while melanoma was overfunded, dermatitis and acne vulgaris were underfunded [10]. However, a focused analysis of these disparities in pediatric dermatology is lacking.

Using the framework established by Hagstrom et al. [10], this study compares NIH funding from 2024 to 2025 with the 10 most burdensome U.S. skin diseases among patients under 20 years. Burden is measured by DALYs published in the updated GBD data (May 2024). Addressing these funding gaps will enhance understanding and innovation in pediatric dermatology, improving long‐term health and well‐being.

## 2 Materials and Methods

This cross‐sectional analysis compared the 10 most burdensome GBD 2021 skin conditions in pediatric populations with total NIH grant funding (2024–2025), as determined by NIH RePORTER. The GBD 2021 examined 18 skin conditions or groupings: acne vulgaris, alopecia areata, atopic dermatitis, contact dermatitis, seborrheic dermatitis, basal cell carcinoma, squamous cell carcinoma, malignant melanoma, cellulitis, decubitus ulcer, pruritus, psoriasis, pyoderma, scabies, urticaria, fungal skin diseases, viral skin diseases, and “other skin and subcutaneous diseases.” DALYs were expressed as a percentage of total U.S. DALYs using the GBD Results Tool from the Institute for Health Metrics and Evaluation. Only U.S.‐specific data were used, adjusted for pediatric populations (< 20 years). Parameters selected included “Cause of death or injury,” “DALYs,” “Percent,” “United States,” “< 20 years,” “Both” for sex, and “2021.”

The resulting top 10 skin conditions by DALYs were: acne vulgaris, atopic dermatitis, contact dermatitis, cellulitis, pruritus, psoriasis, urticaria, fungal skin diseases, viral skin diseases, and “other skin and subcutaneous diseases.” These were then entered into the GBD 2021 Methods Appendices to identify corresponding ICD‐10 codes, which were used to generate search terms via ICD10Data.com. For instance, ICD‐10:L20 (atopic dermatitis) yielded terms like “Infantile eczema” and “Atopic neurodermatitis.”

NIH grants from 2024 and 2025 were identified using the NIH RePORTER Advanced Projects Search tool. Parameters included: “Fiscal Year” = 2024 and 2025, “United States,” “Project Title,” and “Abstracts,” and ICD‐10‐based search terms combined with Boolean logic. Results were downloaded as Excel files.

Grant titles and abstracts were evaluated for relevance and pediatric focus. Only grants explicitly focused on the condition were included. Mentions in “project terms,” “application,” or “public health relevance” were excluded from categorization. Abstracts were labeled “Yes” or “No” for inclusion. Duplicates were removed.

The number of pediatric‐specific grants was also recorded. To be categorized as pediatric‐focused, a grant had to explicitly mention a pediatric population, such as children, adolescents, or specific age groups under 20 years old, in either the title or abstract. If the age group studied was not specified, or if the project was a basic science study without human subjects, it was not considered pediatric‐specific. This approach reflects the distinct pathophysiology, clinical presentation, and treatment considerations in pediatric dermatology, where lack of age‐specific mention may indicate limited applicability to this population.

For each condition, total funding was calculated by summing awarded amounts from included grants. This total was used to compute the percentage of NIH funding ($47.44 billion) allocated to each condition. Conditions were ranked by NIH funding percentage and compared with their DALY metrics.

Data were extracted and categorized by two authors (N.A. and W.A.) in December 2024, with consensus review by a senior author (E.M.) to resolve discrepancies. Pediatric‐specific NIH funding and grant counts were matched to each condition's U.S. DALY metric. A qualitative analysis identified overfunded and underfunded conditions relative to burden.

## 3 Results

Between 2024 and 2025, the NIH supported 307 grants across the 10 most burdensome pediatric skin conditions, totaling $388 million, representing 0.98% of NIH‐issued grants and 0.77% of NIH funding. Comparison of DALYs and funding revealed that psoriasis, cellulitis, pruritus, and other skin/subcutaneous diseases were overfunded relative to burden, while atopic dermatitis, viral skin diseases, acne vulgaris, urticaria, and fungal skin diseases were underfunded. Contact dermatitis was appropriately matched.

Atopic dermatitis received the largest proportion of NIH dermatology‐related funding ($62.99 M, 13.28%) and the highest number of grants (98), with 47 of these grants (48%) explicitly focusing on pediatric populations. Psoriasis was second in funding ($29.95 M, 6.31%) with 71 total grants; but only 3 (4.2%) focused on pediatric patients. Other skin and subcutaneous diseases received the third‐highest allocation ($39.48 M, 8.32%, 100 grants), but just 3 grants (3%) included a pediatric focus.

Notably, viral skin diseases ranked second in disease burden but received only $2.35 M (0.50%) across 2 grants, none of which explicitly focused on pediatrics. Acne vulgaris received $16.86 M (3.56%) across 16 grants, but only 2 (12.5%) were pediatric‐specific. Urticaria was similarly underrepresented, with only 1 of 9 grants (11.1%) focusing on pediatric populations. Fungal skin diseases received no dedicated NIH funding or grants.

This pediatric‐specific analysis highlights a critical gap: even among dermatologic conditions with substantial pediatric burden, the majority of NIH‐funded research grants did not include a clear pediatric focus.

## 4 Discussion

This study reveals a persistent misalignment between NIH funding and the burden of pediatric skin diseases in the U.S. in both total dollars allocated and in the lack of pediatric‐focused research. While some conditions such as atopic dermatitis received funding somewhat aligned with their burden, others, particularly viral and fungal skin diseases, acne vulgaris, and urticaria, remained significantly underfunded both in total support and in pediatric inclusion (Table 1).

**TABLE 1:** Categorization of National Institutes of Health grants, funding, and US Global Burden of Disease disability‐adjusted life year metrics.

Category	ICD‐10 codes populating disease category in GBD	Funding^a^ (percent)^b^	No. of NIH grants in 2024/2025	No. of NIH grants in 2024/2025 with pediatric focus	US DALY 2021 absolute no.^c^	US DALY 2021 skin disease rank^d^	NIH funding 2024/2025 rank
Atopic dermatitis	L20	$62,994,904.00 (13.28)	98	47	2.15	1	1
Viral skin diseases	B07, B08.1	$2,354,686.00 (0.50)	2	0	1.93	2	6
Acne vulgaris	L70 (excluding L70.4)	$16,863,364.00 (3.56)	16	2	1.17	3	4
Urticaria	L50	$8,431,682.00 (1.78)	9	1	0.77	4	5
Psoriasis	L40, L41	$29,949,887.00 (6.31)	71	3	0.44	5	3
Other skin and subcutaneous diseases	B08 (excluding B08.1), B09, B85, B87‐88, D86.3, E80.1‐80.2, L10‐13, L28, L42‐44, L49, L51‐53, L56‐57, L59, L60, L64‐68, L72‐75, L81‐87, L90‐92, L94‐95	$39,480,498.00 (8.32)	100	3	0.29	6	2
Contact dermatitis	L22‐26	$1,921,034.00 (0.41)	4	0	0.21	7	7
Fungal skin diseases	B35.2‐35.6, B35.9	$0.00 (0.00)	0	0	0.08	8	10
Cellulitis	L03	$1,773,505.00 (0.37)	4	1	0.05	9	8
Pruritus	L29	$1,481,605.00 (0.31)	3	0	0.05	10	9

*Note*: Arranged in order of decreasing US DALY.

^a^ For fiscal years 2024 and 2025.

^b^ Total funding for all NIH skin categories is $47,439,000,000.00.

^c^ All ages.

^d^ Of 18 skin condition categories studied by GBD 2021.

Further, only 47.9% of atopic dermatitis grants included a pediatric focus, despite its leading burden in children. Psoriasis, although well‐funded overall, had a pediatric focus in just 4.2% of grants. Viral skin diseases and fungal skin diseases, both high‐burden in children, received almost no dedicated pediatric research. These trends suggest that even when funding is directed toward a relevant condition, pediatric populations are often underrepresented or overlooked in NIH‐supported research [4, 7, 9].

Overall, given the long‐term physical, psychological, and social implications of pediatric skin disease, findings from this study underscore the urgent need for more research explicitly designed for and inclusive of pediatric patients. Without such targeted funding, gaps in understanding and care are likely to persist, limiting the development of age‐appropriate therapies and interventions [9].

This study has several limitations. First, while DALYs provide a useful standardized measure of disease burden, they are only one of many factors influencing NIH funding. Historical funding patterns, research feasibility, and the unpredictable nature of scientific discovery all contribute to funding decisions. For example, psoriasis is now well‐funded not solely due to burden but because earlier discoveries enabled large‐scale translational research and lasting strong research infrastructure [11]. Further, much of basic science research operates several steps removed from clinical endpoints, often informing unexpected conditions. For example, dupilumab, initially developed for atopic dermatitis, later showed promise in pediatric alopecia areata by targeting shared immunopathogenesis [12]. Given these complexities, perfect alignment between burden and funding is neither expected nor always appropriate. However, identifying where substantial gaps remain, particularly for pediatric populations, can help guide more equitable prioritization.

Methodologically, the study relies on GBD 2021 data, which may underrepresent rare or underserved pediatric conditions [13]. Second, NIH RePORTER may not capture the full scope of pediatric dermatology research due to variability in grant categorization and keyword usage. A systematic approach based on ICD‐10 codes was used to minimize this issue, but omissions remain possible.

Additionally, filtering grants based on explicit mention of pediatric populations may underestimate funding relevant to children. Some grants applicable to pediatric dermatology may not have specified a pediatric focus in titles or abstracts, even if their findings are ultimately generalizable to or inclusive of pediatric patients. This limitation could lead to an underrepresentation of pediatric‐focused research in our analysis. Finally, the analysis also covers only two fiscal years (2024–2025), which may not reflect broader funding trends.

It is important to acknowledge that NIH funding priorities are influenced by broader sociopolitical contexts, which can lead to mismatches between burden and funding [9]. Rapid shifts in funding structure and emphasis can occur in response to changes in administration or public policy. While our study does not address this directly, sustained federal support remains essential for advancing pediatric dermatology research and addressing unmet needs. Future research should explore the underlying drivers of funding misalignment in pediatric dermatology, including the influence of advocacy, public awareness, and political priorities.

Studies assessing how funding gaps affect patient outcomes, such as access to care and treatment innovation, are also warranted. The GBD framework can be used to inform more burden‐aligned NIH funding strategies, and stakeholders should advocate for increased transparency and targeted pediatric dermatology grants. Longitudinal tracking of funding trends relative to disease burden will be essential to evaluate the effectiveness of these efforts.

## Conflicts of Interest

The authors declare no conflicts of interest.

## Data Availability

The data that support the findings of this study are available from the corresponding author upon reasonable request.
